# Supplementary material for: Nlrc3 signaling is indispensable for hematopoietic stem cell emergence via Notch signaling in vertebrates
Source: Nat Commun. 2024 Jan 3;15:226. doi: 10.1038/s41467-023-44251-6 (PMC10764762; doi:10.1038/s41467-023-44251-6)
Supplement: Supplementary file 1 — Supplementary Information [file 41467_2023_44251_MOESM1_ESM.pdf]

Supplementary Information

Nlrc3 signaling is indispensable for hematopoietic stem cell emergence via Notch signaling in vertebrates

Shuyang Cai. et al

Corresponding authors:

*He Huang

Professor/MD/Ph.D.

Bone Marrow Transplantation Center, The First Affiliated Hospital, School of Medicine,

E-mail: huanghe@zju.edu.cn

*Pengxu Qian

Investigator

School of Medicine, Zhejiang University

Email: axu@zju.edu.cn

*Jiahui Lu

Professor

Shanghai Municipal Hospital of Traditional Chinese Medicine, Shanghai University of Traditional Chinese Medicine

E-mail: lujiahui_sci@126.com

The PDF file includes:

Supplementary Figure 1 to 10

Supplementary Table 1 to 5

Supplementary Figures

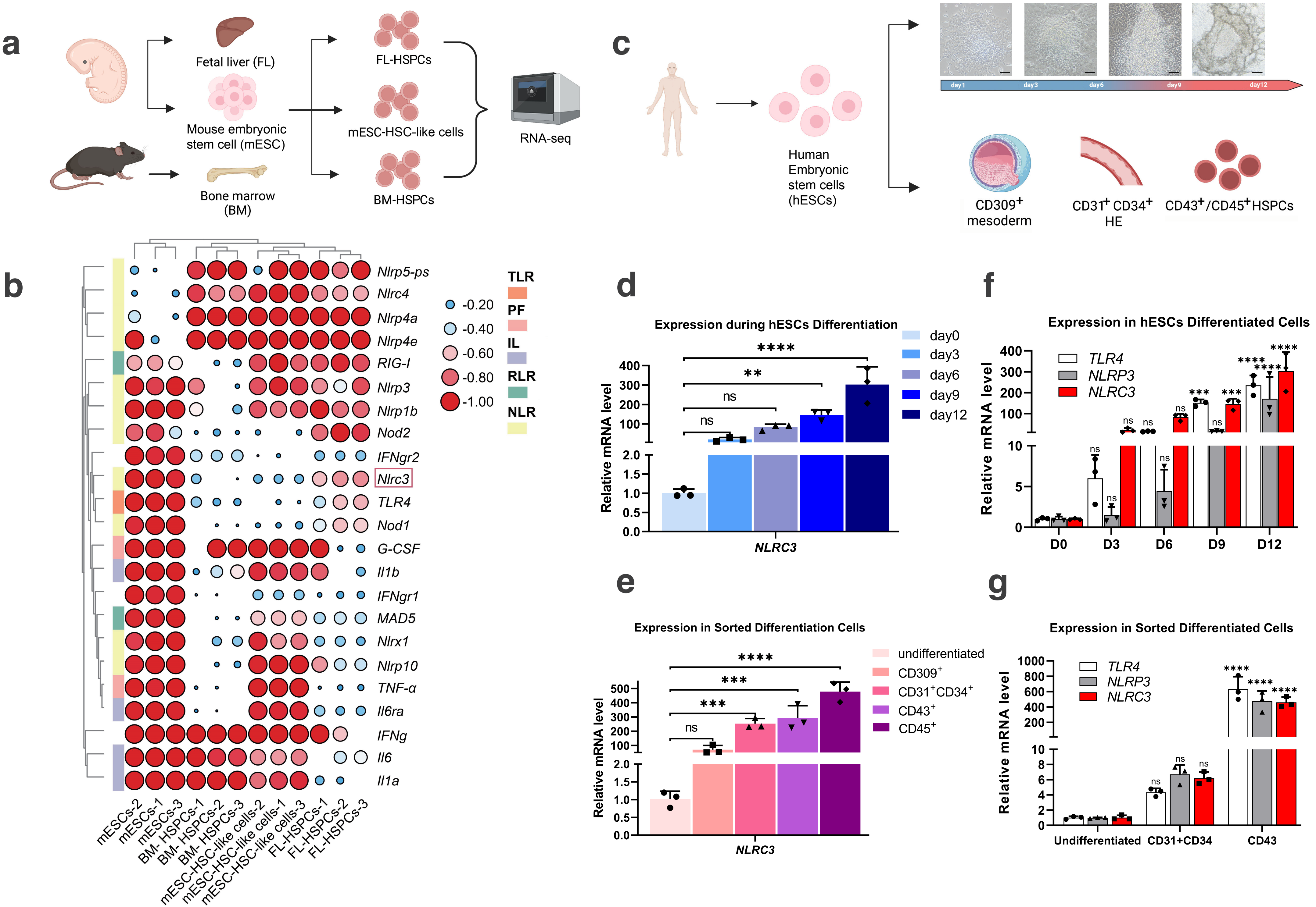
a
Fetal liver (FL)
Mouse embryonic stem cell (mESC)
Bone marrow (BM)
FL-HSPCs
mESC-HSC-like cells
BM-HSPCs
RNA-seq
b
Nlrp5-ps
Nlrc4
Nlrp4a
Nlrp4e
RIG-I
Nlrp3
Nlrp1b
Nod2
IFNgr2
Nlrc3
TLR4
Nod1
G-CSF
Il1b
IFNgr1
MAD5
Nlrx1
Nlrp10
TNF-α
Il6ra
IFNg
Il6
Il1a
-0.20
-0.40
-0.60
-0.80
-1.00
TLR
PF
IL
RLR
NLR
mESCs-2
mESCs-1
mESCs-3
BM- HSPCs-1
BM- HSPCs-2
BM- HSPCs-3
mESC-HSC-like cells-2
mESC-HSC-like cells-1
mESC-HSC-like cells-3
FL-HSPCs-1
FL-HSPCs-2
FL-HSPCs-3
c
Human Embryonic stem cells (hESCs)
day1
day3
day6
day9
day12
CD309+ mesoderm
CD31+ CD34+ HE
CD43+/CD45+HSPCs
d
Expression during hESCs Differentiation
day0
day3
day6
day9
day12
Relative mRNA level
NLRC3
ns
ns
**
****
e
Expression in Sorted Differentiation Cells
undifferentiated
CD309+
CD31+CD34+
CD43+
CD45+
Relative mRNA level
NLRC3
ns
***
***
****
f
Expression in hESCs Differentiated Cells
TLR4
NLRP3
NLRC3
Relative mRNA level
D0
D3
D6
D9
D12
g
Expression in Sorted Differentiated Cells
TLR4
NLRP3
NLRC3
Relative mRNA level
Undifferentiated
CD31+CD34
CD43

Supplemental Figure 1. *NLRC3* is highly expressed in induced HSPC differentiated from mouse ESCs or human ESCs.

a Schematic overview of mouse embryonic stem cells (ESCs) sorted for culture in various differentiation systems. **b** Bubble plot of sequencing data demonstrating the expression of nucleotide-binding domain leucine-rich repeat (NLR) family members including *Nlrc3* and related inflammatory genes as mouse ESCs matured into hematopoietic differentiation in various cell types, including undifferentiated ESCs, ESCs derived LSKCD201^{+} HSC-like cells, fetal liver (FL) derived HSPCs and bone marrow (BM) derived HSPCs. **c** Schematic overview of the chemical defined system to induce HSPCs from hESCs. **d** Dynamic analysis of *NLRC3* expression by qPCR during hematopoietic differentiation of hESCs. Relative expression is normalized to the expression level of undifferentiated hESCs. ns = 0.9740, 0.1579, **P = 0.0084, ****P < 0.0001. **e** qPCR analysis of *NLRC3* expression in undifferentiated hESCs, mesoderm (APLNR^{+}), HE (CD31^{+} and CD34^{+}), and HSPCs (CD43^{+} and CD45^{+}) derived from hESCs. Relative expression normalized to the expression level in undifferentiated hESCs. ns = 0.4688, ***P = 0.0007, 0.0002, ****P < 0.0001. **f** Dynamic expression of *NLRC3, NLRP3,* and *TLR4* by qPCR during hematopoietic differentiation of hESCs. Relative expression is normalized relative to undifferentiated hESCs. ns = 0.9999, 0.9918, 0.9734, 0.0986, 0.9907， > 0.9999, ***P = 0.0004, 0.0007, ****P < 0.0001. **g** qPCR analysis of *NLRC3, NLRP3,* and *TLR4* in undifferentiated hESCs, HE (CD31^{+} and CD34^{+}), and HSPCs (CD43^{+}) derived from hESCs. Relative expression is normalized to the expression levels of undifferentiated hESCs. ns = 0.9983, 0.9949, 0.9958, ****P < 0.0001. Error bars, mean ± s.d., the exact p-values mentioned above are listed from left to right, by using one-way ANOVA – Sidak test in **d, e,** two-way ANOVA with Tukey's post hoc test in **f, g.** *n* = 3 biological replicates in **d, e, f, g**. Source data are provided as a Source Data file.

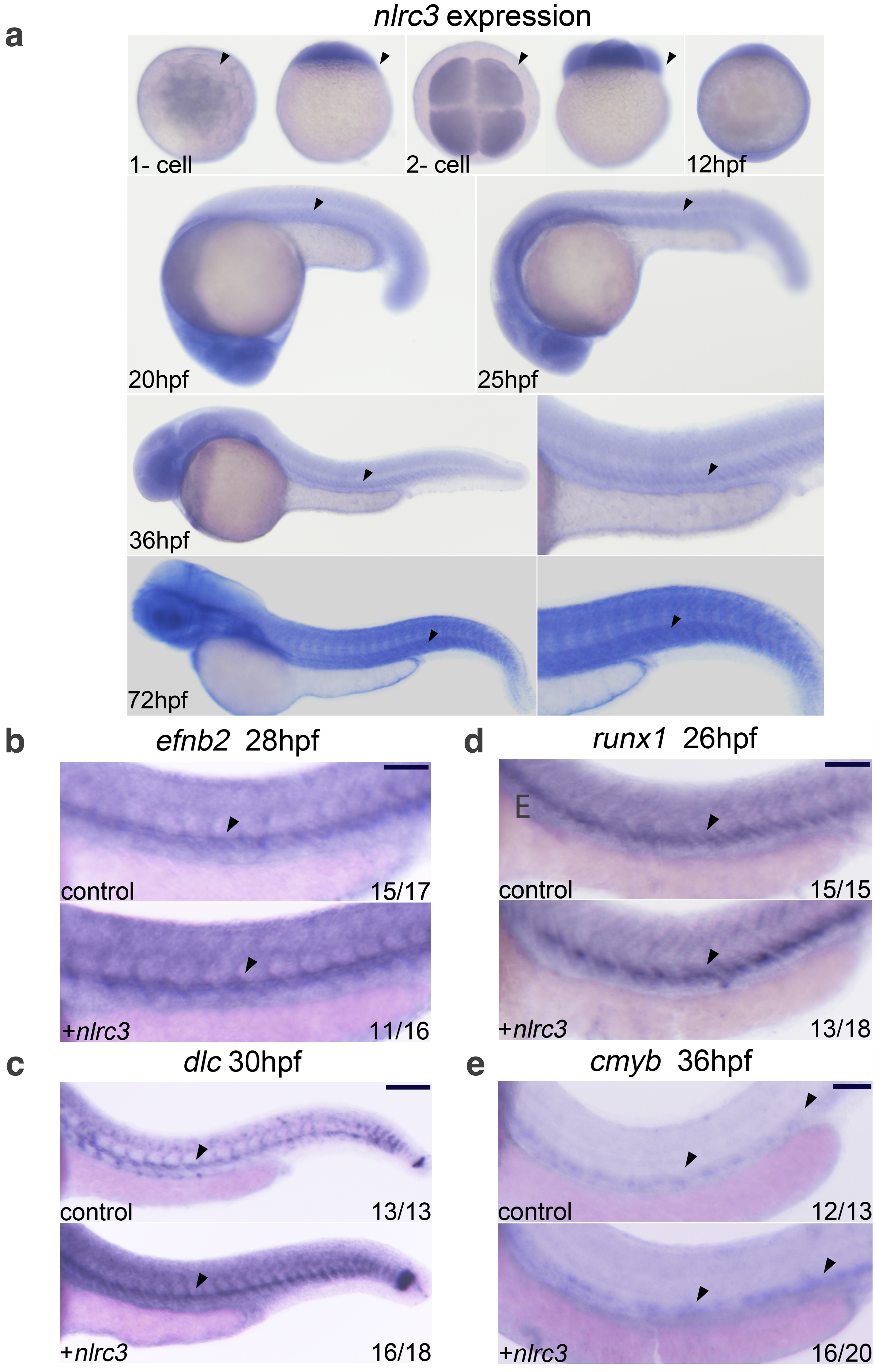

a
nlrc3 expression
1- cell
2- cell
12hpf
20hpf
25hpf
36hpf
72hpf
b
efnb2 28hpf
control
15/17
+nlrc3
11/16
c
dlc 30hpf
control
13/13
+nlrc3
16/18
d
runx1 26hpf
E
control
15/15
+nlrc3
13/18
e
cmyb 36hpf
control
12/13
+nlrc3
16/20

Supplemental Figure 2. The expression of *nlrc3* pattern in zebrafish.

a Expression pattern of *nlrc3* during zebrafish embryogenesis: The stage examined by whole-mount in situ hybridization (WISH) is shown in each panel: 1 - cell stage, 4 - cell stage, 12 hours postfertilization (hpf), 20 hpf (Scale bars: 200 μ m), 25 hpf, 36 hpf and 72 hpf. **b** Expression of arterial marker *efnb2* in control embryos and control embryos with co-expression of *nlrc3* probe at 28 hpf by WISH. **c** Expression of arterial marker *dlc* in control embryos and control embryos with co-expression of *nlrc3* probe at 30 hpf by WISH. **d** Expression of HSPC marker *runx1* in control embryos and control embryos with co-expression of *nlrc3* probe at 26 hpf by WISH. **e** Expression of HSPC marker *cmyb* in control embryos and control embryos with co-expression of *nlrc3* probe at 36 hpf by WISH. Scale bars, 100 μm in **b**, **c**, **d**, **e**. Source data are provided as a Source Data file.

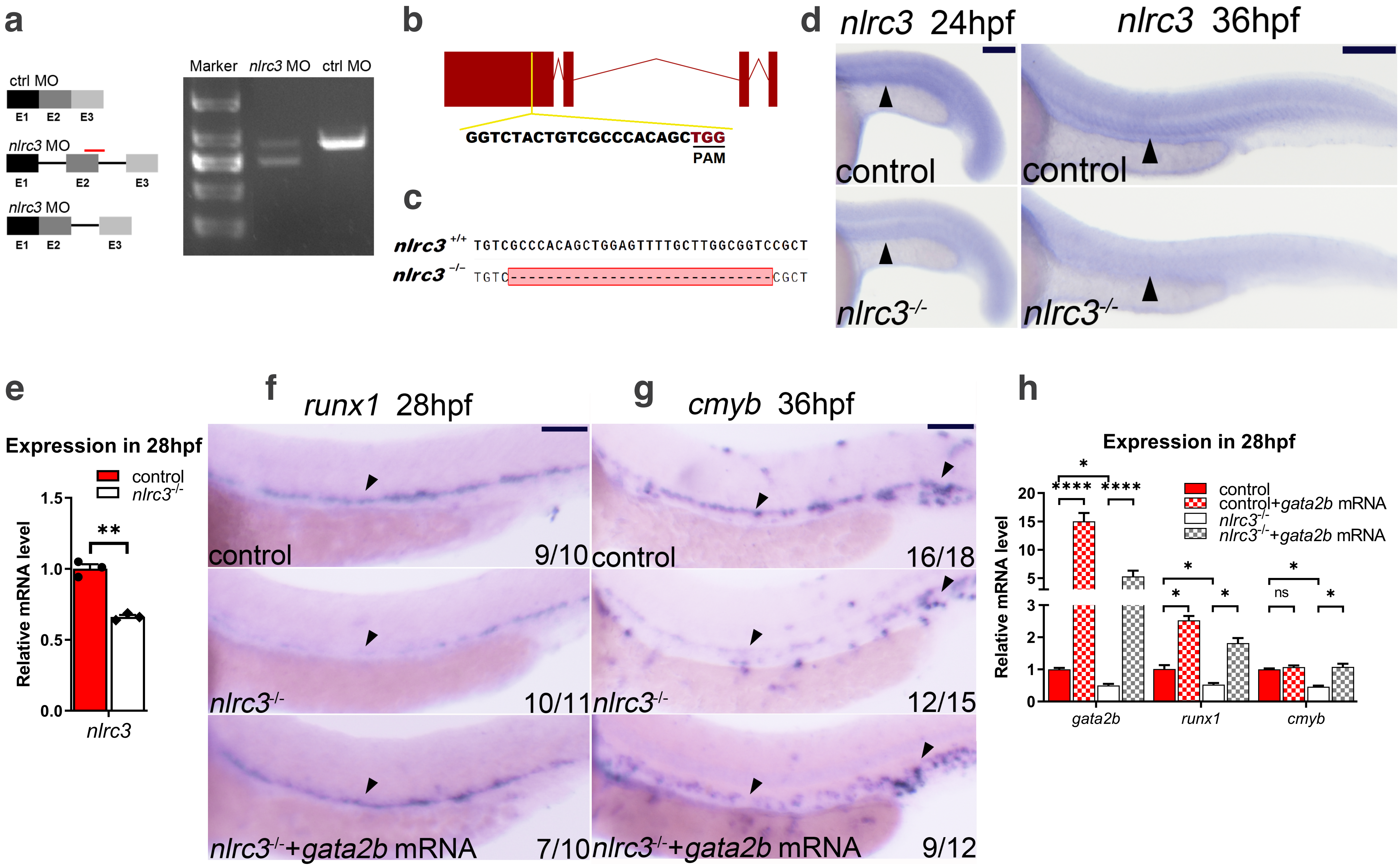

a
ctrl MO
E1 E2 E3
nlrc3 MO
E1 E2 E3
nlrc3 MO
E1 E2 E3
Marker nlrc3 MO ctrl MO
b
GGTCTACTGTCGCCCACAGCTGG
PAM
c
nlrc3 +/+ TGTCGCCCACAGCTGGAGTTTTGCTTGGCGGTCCGCT
nlrc3 -/- TGTC-----------------------------CGCT
d
nlrc3 24hpf
nlrc3 36hpf
control
control
nlrc3-/-
nlrc3-/-
e
Expression in 28hpf
control
nlrc3-/-
Relative mRNA level
1.5
1.0
0.5
0.0
**
nlrc3
f
runx1 28hpf
control 9/10
nlrc3-/- 10/11
nlrc3-/-+gata2b mRNA 7/10
g
cmyb 36hpf
control 16/18
nlrc3-/- 12/15
nlrc3-/-+gata2b mRNA 9/12
h
Expression in 28hpf
control
control+gata2b mRNA
nlrc3-/-
nlrc3-/-+gata2b mRNA
Relative mRNA level
20
15
10
5
3
2
1
0
* **** ****
* * *
* ns *
gata2b runx1 cmyb

Supplemental Figure 3. Generation and validation of *nlrc3* morphants and mutants.

a Schematic diagram of splice morpholino knockdown target in second exon of *nlrc3*. **b** The panel showing the target site in the second exon of *nlrc3* designed for the CRISPR–Cas9 mutants. **c** The panel revealing the 29 bp deletion in the *nlrc3* target site. **d** The expression of *nlrc3* in the dorsal aorta (DA) during EHT stage of the WT and *nlrc3* mutants. **e** Expression of *nlrc3* in control and *nlrc3* mutants at 28 hpf by qPCR. **P = 0.0012. **f** Expression of HSPC marker *runx1* in control embryos, *nlrc3* mutants, *nlrc3* mutants with overexpression of *gata2b* mRNA at 28 hpf by WISH. **g** Expression of HSPC marker *cmyb* in control embryos, *nlrc3* mutants, *nlrc3* mutants with overexpression of *gata2b* mRNA at 36 hpf by WISH. **h** Expression of *gata2b*, *runx1* and *cmyb* in control, control with overexpression of *gata2b* mRNA, *nlrc3* mutants, and *nlrc3* mutants with overexpression of *gata2b* mRNA at 28 hpf by qPCR. ns = 0.9997, *P = 0.0418, 0.0261, 0.0213, 0.0204, 0.0133, 0.0155, ****P < 0.0001. Error bars, mean ± s.d., the exact p-values mentioned above are listed from left to right, by using two-tailed, unpaired student's *t*-test in **e**, **h**, *n* = 3 biological replicates in **e, h**. Source data are provided as a Source Data file.

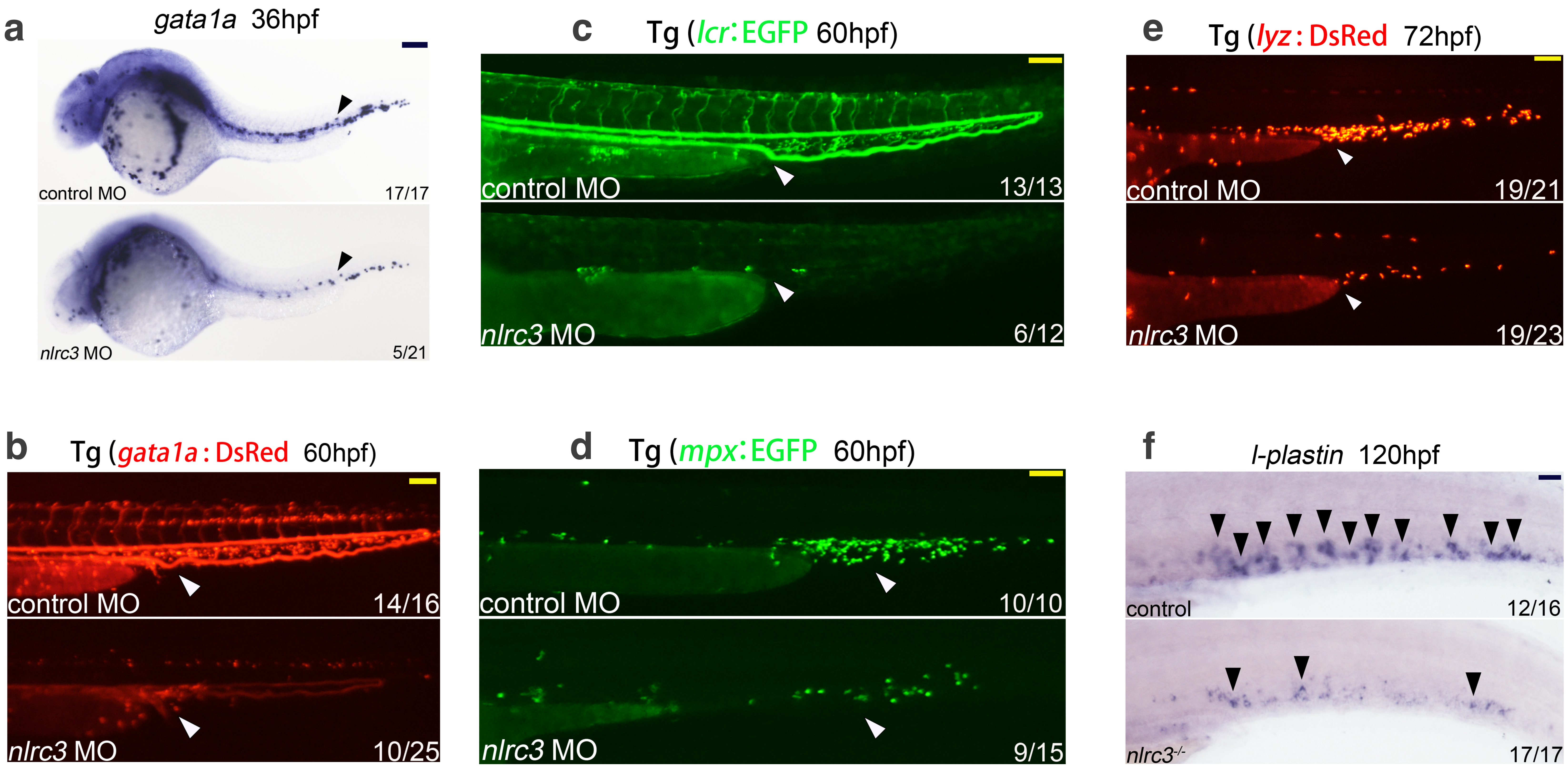

a
gata1a 36hpf
control MO
17/17
nlrc3 MO
5/21
b
Tg (gata1a : DsRed 60hpf)
control MO
14/16
nlrc3 MO
10/25
c
Tg (lcr: EGFP 60hpf)
control MO
13/13
nlrc3 MO
6/12
d
Tg (mpx: EGFP 60hpf)
control MO
10/10
nlrc3 MO
9/15
e
Tg (lyz : DsRed 72hpf)
control MO
19/21
nlrc3 MO
19/23
f
l-plastin 120hpf
control
12/16
nlrc3-/-
17/17

Supplement Figure 4. *Nlrc3* signaling is imperative for HSPC differentiation in zebrafish.

a Expression of erythroid marker *gata1a* in control embryos and *nlrc3* morphants at 36 hpf by WISH. **b** Snapshot of erythroid marker Tg (*gata1a*:DsRed) in control embryos and *nlrc3* morphants at 60 hpf. **c** Snapshot of erythroid marker Tg (*lcr*:EGFP) in control embryos and *nlrc3* morphants at 60 hpf. **d** Snapshot of myeloid marker Tg (*mpx*:EGFP) in control embryos and *nlrc3* morphants at 60 hpf. **e** Snapshot of myeloid marker Tg (*lyz*:DsRed) in control embryos and *nlrc3* morphants at 72 hpf. **f** Expression of myeloid marker *l-plastin* in control embryos and *nlrc3* mtants at 120 hpf by WISH. Each sample was composed of at least 5 embryos and Scale bars, 100 μm in **a**, **b**, **c**, **d, e, f**. Source data are provided as a Source Data file.

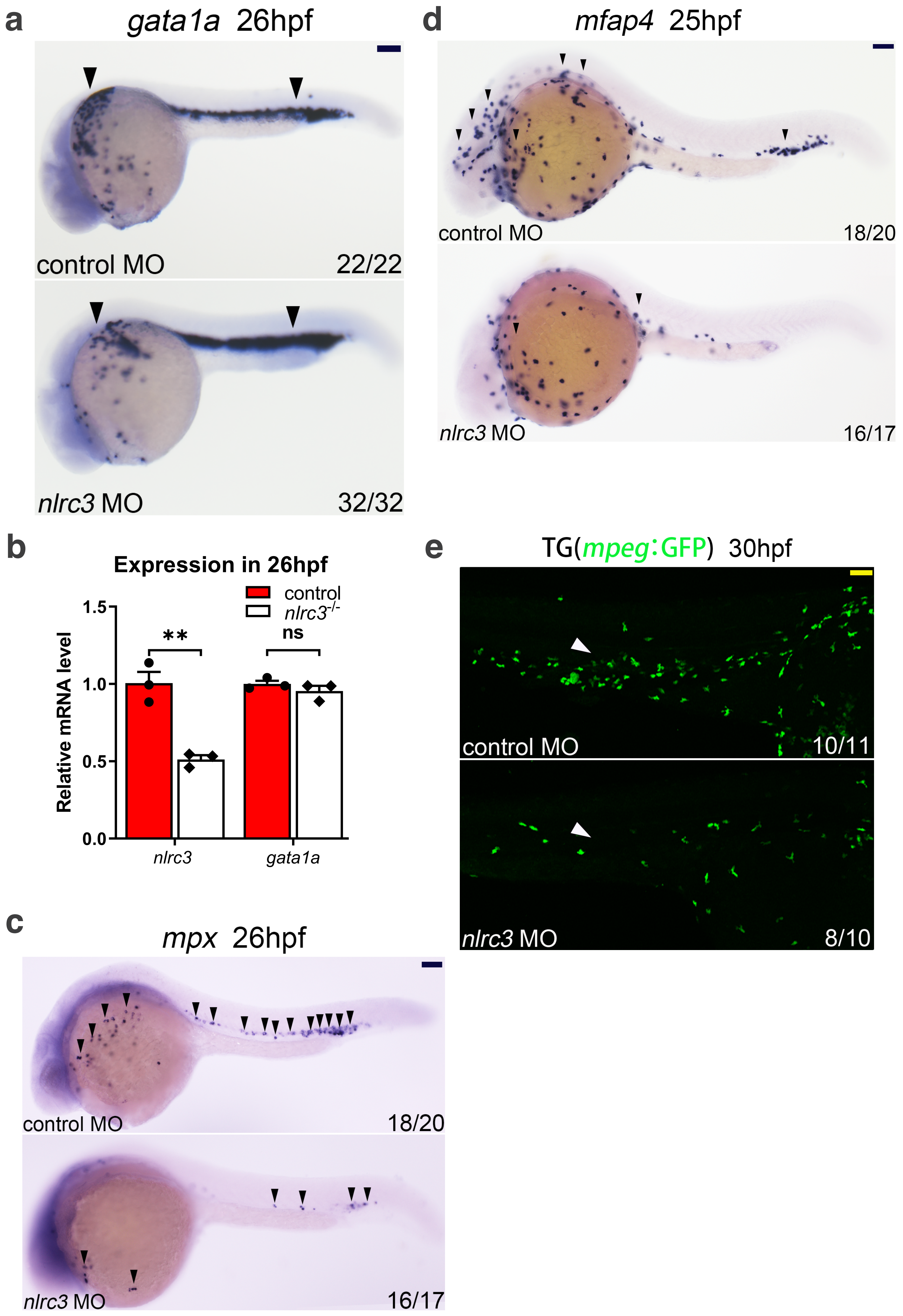
a
gata1a 26hpf
control MO
22/22
nlrc3 MO
32/32
b
Expression in 26hpf
control
nlrc3-/-
Relative mRNA level
1.5
1.0
0.5
0.0
**
ns
nlrc3
gata1a
c
mpx 26hpf
control MO
18/20
nlrc3 MO
16/17
d
mfap4 25hpf
control MO
18/20
nlrc3 MO
16/17
e
TG(mpeg:GFP) 30hpf
control MO
10/11
nlrc3 MO
8/10

Supplement Figure 5. ***Nlrc3*** **signaling was indispensable for primitive neutrophils and macrophages but not erythropoiesis.**

a Expression of erythroid marker *gata1a* in control embryos and *nlrc3* morphants at 26 hpf by WISH. **b** Expression of erythroid gene *gata1a* in control embryos and *nlrc3* mutants at 26 hpf by qPCR. ns = 0.4808, **P = 0.0075. Error bars, mean ± s.d., by using two-tailed, unpaired student's *t*-test. *n* = 3 biological replicates. **c** Expression of myeloid marker *mpx* in control embryos and *nlrc3* morphants at 26 hpf by WISH. **d** Expression of macrophage marker *mfap4* in control embryos and *nlrc3* morphants at 25 hpf by WISH. **e** Confocal imaging showing the macrophage marker *mpeg*:GFP in control embryos and *nlrc3* morphants at 30 hpf. Scale bars, 100 µm and each sample was composed of at least 5 embryos in **a**, **c**, **d**, **e**. Source data are provided as a Source Data file.

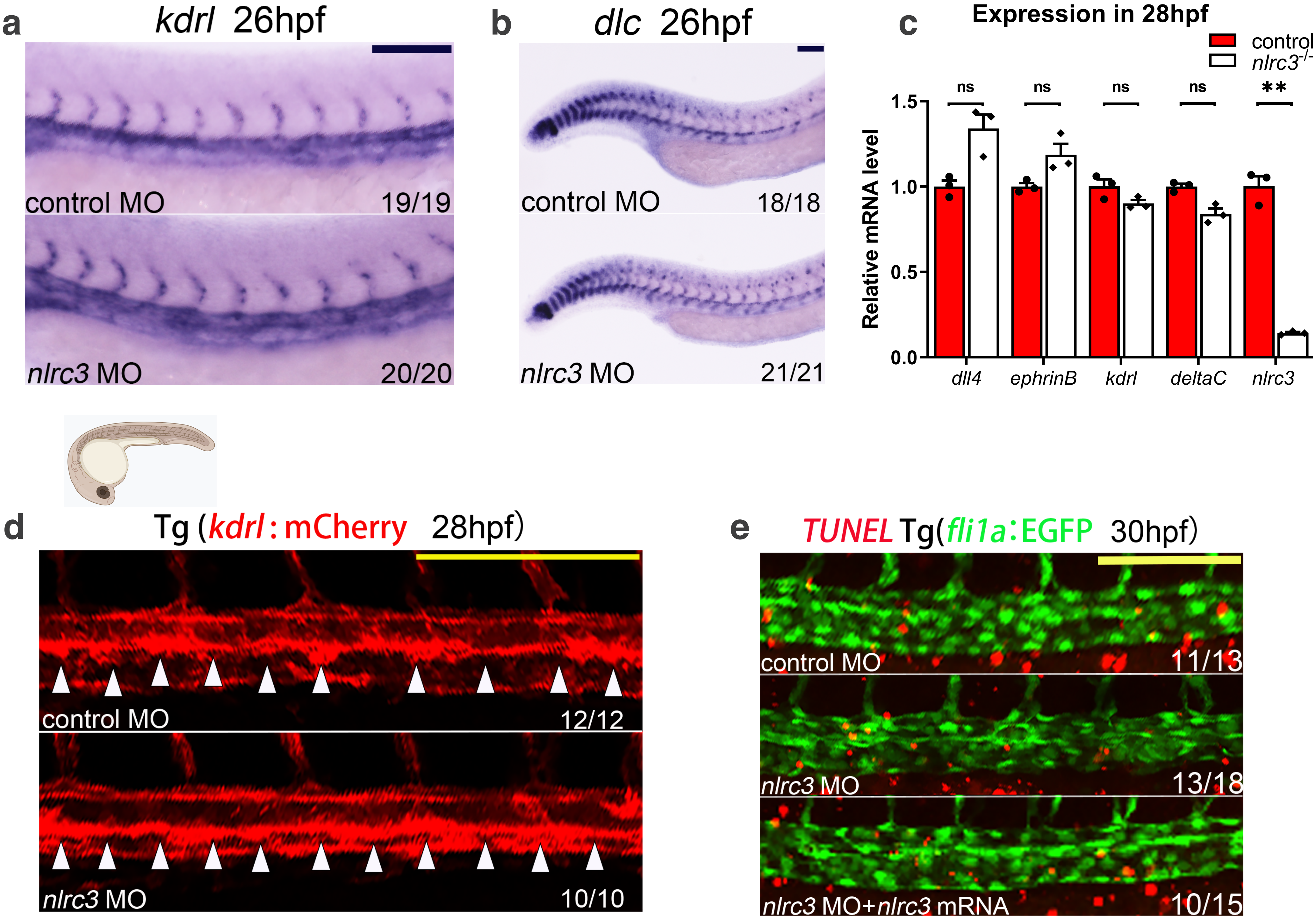

a
kdrl 26hpf
control MO
19/19
nlrc3 MO
20/20
b
dlc 26hpf
control MO
18/18
nlrc3 MO
21/21
c
Expression in 28hpf
control
nlrc3-/-
Relative mRNA level
1.5
1.0
0.5
0.0
ns
ns
ns
ns
**
dll4
ephrinB
kdrl
deltaC
nlrc3
d
Tg (kdrl : mCherry 28hpf)
control MO
12/12
nlrc3 MO
10/10
e
TUNEL Tg(fli1a:EGFP 30hpf)
control MO
11/13
nlrc3 MO
13/18
nlrc3 MO+nlrc3 mRNA
10/15

Supplement Figure 6. *Nlrc3*-deficient embryos displayed normal vasculogenesis and apoptosis.

a Expression of endothelial marker *kdrl* in control embryos and *nlrc3* morphants at 26 hpf by WISH. **b** Expression of arterial marker *dlc* in control embryos and *nlrc3* morphants at 26 hpf by WISH. **c** Expression of endothelial and arterial gene *dll4, ephrinB2, kdrl, and deltaC* in control embryos and *nlrc3* mutants at 28 hpf by qPCR. ns = 0.0931, 0.1147, 0.2372, 0.0742, **P = 0.0043. Error bars, mean ± s.d., by using two-tailed, unpaired student's *t*-test. *n* = 3 biological replicates. **d** Confocal imaging showing the endothelial marker Tg (*kdrl*:mCherry) in control embryos and *nlrc3* morphants at 28 hpf. **e** Confocal imaging showing the maximum projections of the AGM region of 30 hpf Tg (*fli1a*:EGFP) embryos injected in control embryos and *nlrc3* morphants and assayed for TUNEL (red). Each sample was composed of at least 5 embryos. Scale bars, 100 μm in **a**, **b**, **d**, **e**. Illustrations created with BioRender.com. Source data are provided as a Source Data file.

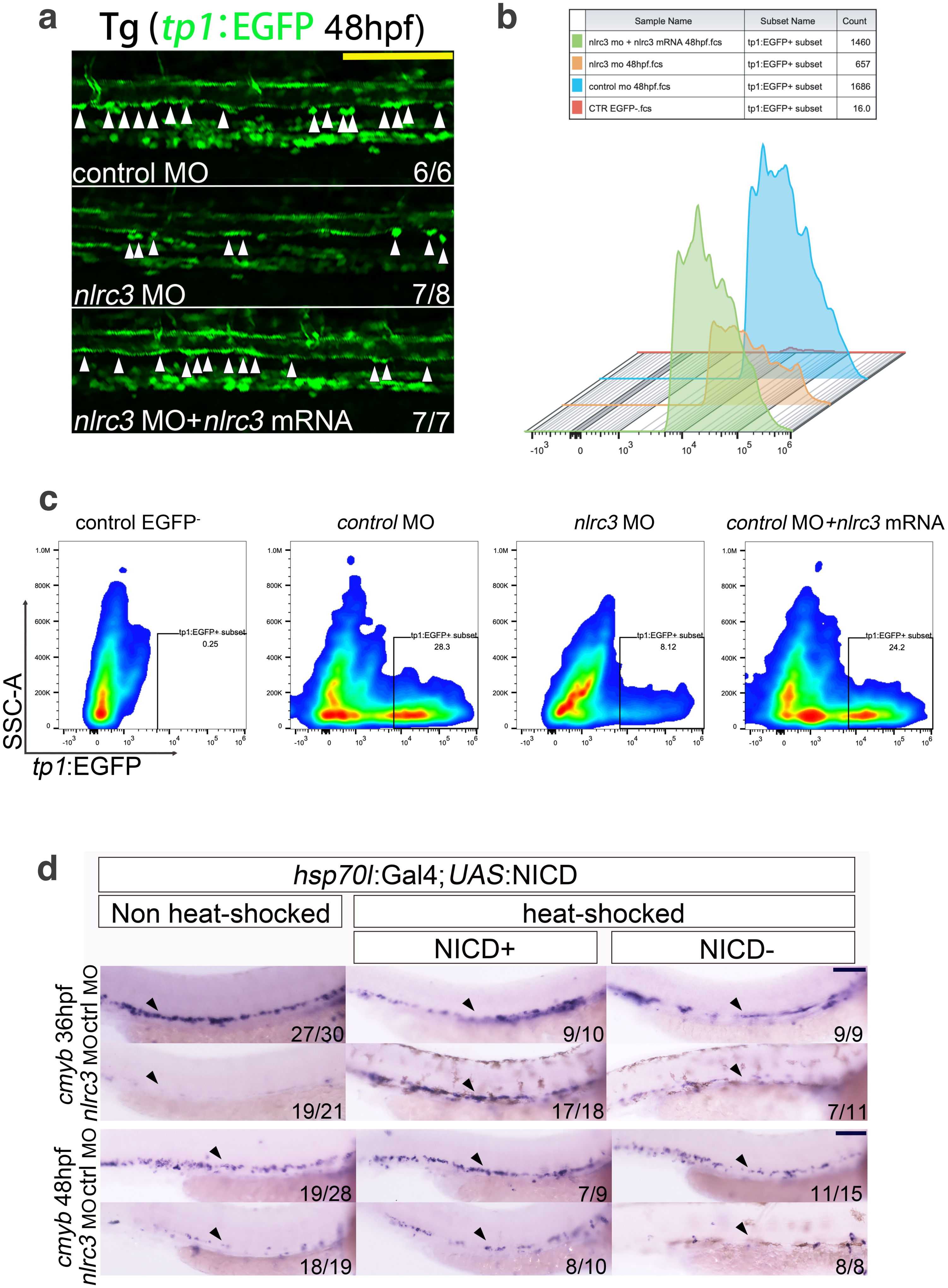
a
Tg (tp1:EGFP 48hpf)
control MO
6/6
nlrc3 MO
7/8
nlrc3 MO+nlrc3 mRNA
7/7
b
Sample Name
Subset Name
Count
nlrc3 mo + nlrc3 mRNA 48hpf.fcs
tp1:EGFP+ subset
1460
nlrc3 mo 48hpf.fcs
tp1:EGFP+ subset
657
control mo 48hpf.fcs
tp1:EGFP+ subset
1686
CTR EGFP-.fcs
tp1:EGFP+ subset
16.0
-10^3
0
10^3
10^4
10^5
10^6
c
control EGFP-
control MO
nlrc3 MO
control MO+nlrc3 mRNA
1.0M
800K
600K
400K
200K
0
tp1:EGFP+ subset
0.25
28.3
8.12
24.2
SSC-A
tp1:EGFP
d
hsp70l:Gal4;UAS:NICD
Non heat-shocked
heat-shocked
NICD+
NICD-
cmyb 36hpf
ctrl MO
nlrc3 MO
27/30
9/10
9/9
19/21
17/18
7/11
cmyb 48hpf
19/28
7/9
11/15
18/19
8/10
8/8

Supplement Figure 7. Notch signaling was in downstream of *nlrc3* during HSPC production.

a Confocal imaging showing the Tg (*tp1*:EGFP) embryos at 48 hpf in the floor of the DA in control embryos, *nlrc3* morphants and *nlrc3* morphants with overexpression of *nlrc3* mRNA. **b** Representative histogram of Tg (*tp1*:EGFP) cells in control embryos, *nlrc3* morphants and *nlrc3* morphants with overexpression of *nlrc3* mRNA at 48 hpf. **c** Flow cytometry results showed the changes of Tg (*tp1*:EGFP) cells in control embryos, *nlrc3* morphants and *nlrc3* morphants with overexpression of *nlrc3* mRNA at 48 hpf. **d** *hsp70*: Gal4; *UAS*:NICD-myc embryos injected with control embryos and *nlrc3* MOs were heat shocked at 18 hpf and WISH for *runx1* was performed at 36 and 48 hpf. Error bars represents means ± SD; Each sample was composed of at least 5 embryos. Scale bars, 100 μm in **a** , **d**. Source data are provided as a Source Data file.

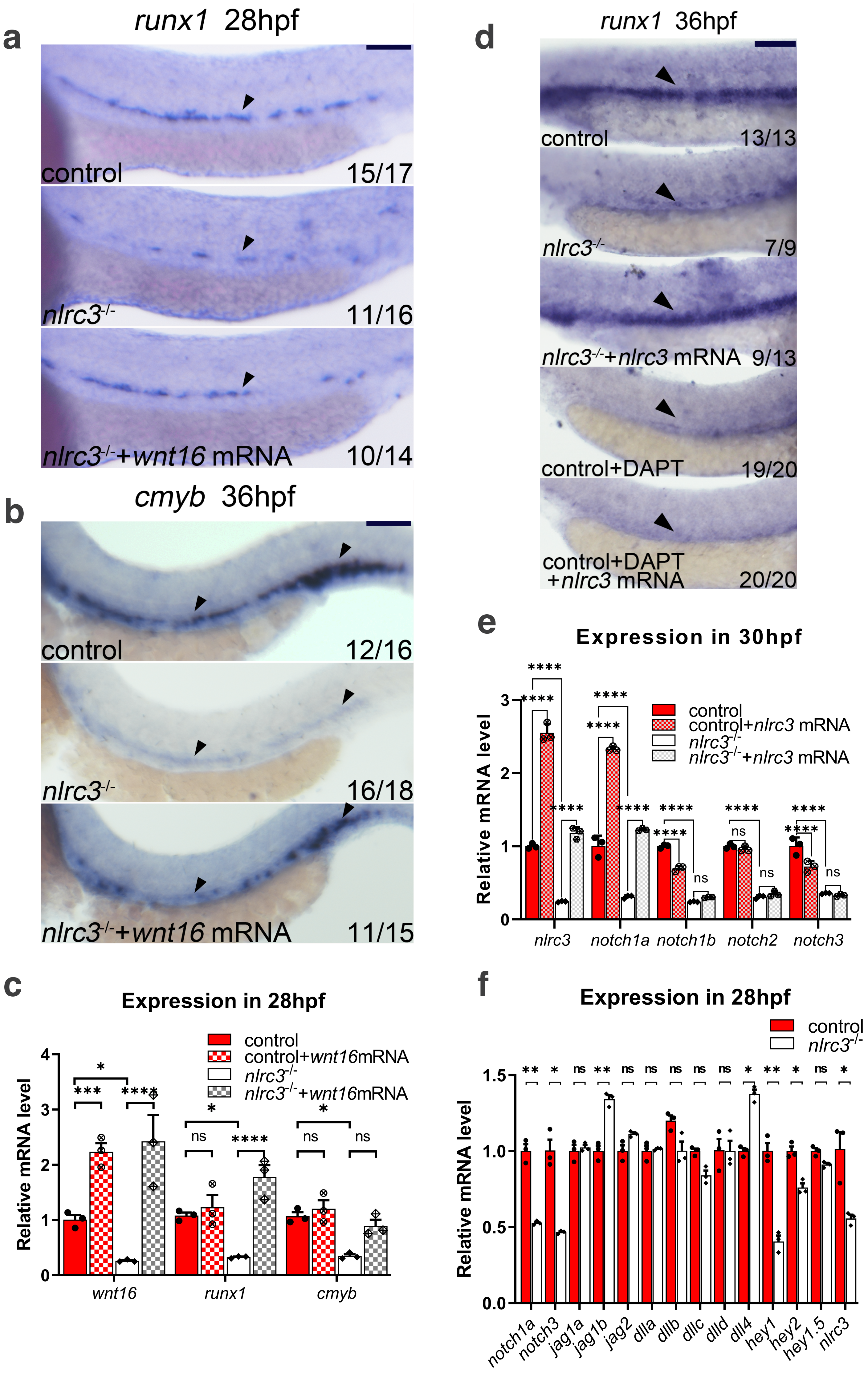

a
runx1 28hpf
control 15/17
nlrc3-/- 11/16
nlrc3-/-+wnt16 mRNA 10/14
b
cmyb 36hpf
control 12/16
nlrc3-/- 16/18
nlrc3-/-+wnt16 mRNA 11/15
c
Expression in 28hpf
control
control+wnt16mRNA
nlrc3-/-
nlrc3-/-+wnt16mRNA
Relative mRNA level
wnt16
runx1
cmyb
d
runx1 36hpf
control 13/13
nlrc3-/- 7/9
nlrc3-/-+nlrc3 mRNA 9/13
control+DAPT 19/20
control+DAPT +nlrc3 mRNA 20/20
e
Expression in 30hpf
control
control+nlrc3 mRNA
nlrc3-/-
nlrc3-/-+nlrc3 mRNA
Relative mRNA level
nlrc3
notch1a
notch1b
notch2
notch3
f
Expression in 28hpf
control
nlrc3-/-
Relative mRNA level
notch1a
notch3
jag1a
jag1b
jag2
dlla
dllb
dllc
dlld
dll4
hey1
hey2
hey1.5
nlrc3

Supplement Figure 8. *Nlrc3* regulated HSPC production via activating the Notch signaling.

a Expression of HSPC marker *runx1* in control embryos, *nlrc3* mutants, *nlrc3* mutants with overexpression of *wnt16* mRNA at 28 hpf by WISH. **b** Expression of HSPC marker *cmyb* in control embryos, *nlrc3* mutants, *nlrc3* mutants with overexpression of *wnt16* mRNA at 36 hpf by WISH. **c** Expression of *wnt16*, *runx1* and *cmyb* in control, control with overexpression of *wnt16* mRNA, *nlrc3* mutants, and *nlrc3* mutants with overexpression of *wnt16* mRNA at 28 hpf by qPCR. ns = 0.9358, 0.9488, 0.1737, *P = 0.0366, 0.0343, 0.0457, ***P = 0.0004, ****P < 0.0001. **d** Expression of HSPC marker *runx1* in control embryos, *nlrc3* mutants, *nlrc3* mutants with overexpression of *nlrc3* mRNA, treatment group with DAPT and treatment group with DAPT and with overexpression of *nlrc3* mRNA at 36 hpf by WISH. **e** Expression of NOTCH genes in control embryos, control with overexpression of *nlrc3* mRNA, *nlrc3* mutants and *nlrc3* mutants with overexpression of *nlrc3* mRNA at 30 hpf by qPCR. ns = 0.5877, 0.8516, 0.8594, 0.9470, ****P < 0.0001. **f** Expression of NOTCH genes in control embryos and *nlrc3* morphants at 28 hpf by qPCR. ns = 0.7233, 0.0518, 0.7827, 0.0730, 0.0742, 0.9558, 0.0771, *P = 0.0120, 0.0198, 0.0121, 0.0417, **P = 0.0071, 0.0028, 0.0046. Error bars, mean ± s.d., by using two-tailed, unpaired student's *t*-test in **f**, two-way ANOVA with Tukey's post hoc test in **c**, **e**, *n* = 3 biological replicates in **c**, **e**, **f**. Scale bars, 100 μm in **a**, **b**, **d**. Source data are provided as a Source Data file.

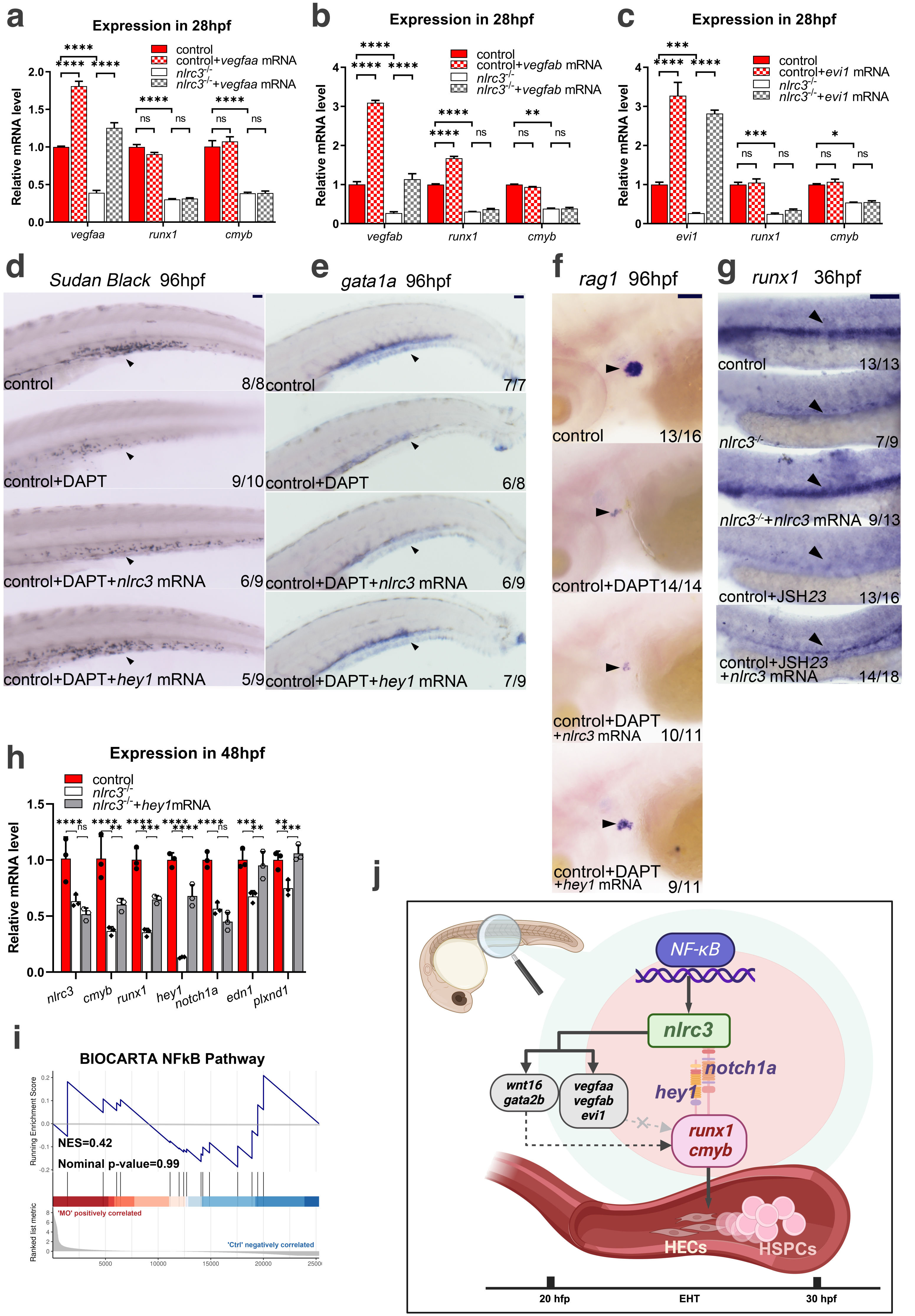

a
Expression in 28hpf
control
control+vegfaa mRNA
nlrc3-/-
nlrc3-/-+vegfaa mRNA
Relative mRNA level
vegfaa
runx1
cmyb
b
Expression in 28hpf
control
control+vegfab mRNA
nlrc3-/-
nlrc3-/-+vegfab mRNA
Relative mRNA level
vegfab
runx1
cmyb
c
Expression in 28hpf
control
control+evi1 mRNA
nlrc3-/-
nlrc3-/-+evi1 mRNA
Relative mRNA level
evi1
runx1
cmyb
d
Sudan Black 96hpf
control 8/8
control+DAPT 9/10
control+DAPT+nlrc3 mRNA 6/9
control+DAPT+hey1 mRNA 5/9
e
gata1a 96hpf
control 7/7
control+DAPT 6/8
control+DAPT+nlrc3 mRNA 6/9
control+DAPT+hey1 mRNA 7/9
f
rag1 96hpf
control 13/16
control+DAPT 14/14
control+DAPT +nlrc3 mRNA 10/11
control+DAPT +hey1 mRNA 9/11
g
runx1 36hpf
control 13/13
nlrc3-/- 7/9
nlrc3-/-+nlrc3 mRNA 9/13
control+JSH23 13/16
control+JSH23 +nlrc3 mRNA 14/18
h
Expression in 48hpf
control
nlrc3-/-
nlrc3-/-+hey1mRNA
Relative mRNA level
nlrc3
cmyb
runx1
hey1
notch1a
edn1
plxnd1
i
BIOCARTA NFkB Pathway
Running Enrichment Score
NES=0.42
Nominal p-value=0.99
'MO' positively correlated
'Ctrl' negatively correlated
Ranked list metric
j
NF-κB
nlrc3
wnt16
gata2b
vegfaa
vegfab
evi1
hey1
notch1a
runx1
cmyb
HECs
HSPCs
20 hfp
EHT
30 hpf

Supplement Figure 9. ***Nlrc3*** **signaling was in the downstream of NF-kB signaling pathway and upstream of** ***hey1*** **for regulating HSPC emergence.**

a Expression of *vegfaa*, *runx1*, and *cmyb* in control, control with overexpression of *vegfaa* mRNA, *nlrc3* mutants, and *nlrc3* mutants with overexpression of *vegfaa* mRNA at 28 hpf by qPCR. ns = 0.4042, 0.9966, 0.6902, > 0.9999, ****P < 0.0001. **b** Expression of *vegfab, runx1, and cmyb* in control, control with overexpression of *vegfab* mRNA, *nlrc3* mutants, and *nlrc3* mutants with overexpression of *vegfab* mRNA at 28 hpf by qPCR. ns = 0.8504, 0.8351, > 0.9999, ****P < 0.0001. **c** Expression of *evi1, runx1, and cmyb* in control, control with overexpression of *evi1* mRNA, *nlrc3* mutants, and *nlrc3* mutants with overexpression of *evi1* mRNA at 28 hpf by qPCR. ns = 0.9903, 0.9143, 0.9685, 0.9999, *P = 0.0346, ***P = 0.0005, 0.0004. **d** Expression of *Sudan Black* in control embryos, treatment group with DAPT, treatment group with DAPT and with overexpression of *nlrc3* mRNA, treatment group with DAPT and with overexpression of *hey1* mRNA at 96 hpf. **e** Expression of *gata1a* in control embryos, treatment group with DAPT, treatment group with DAPT and with overexpression of *nlrc3* mRNA, treatment group with DAPT and with overexpression of *hey1* mRNA at 96 hpf. **f** Expression of *rag1* in control embryos, treatment group with DAPT, treatment group with DAPT and with overexpression of *nlrc3* mRNA, treatment group with DAPT and with overexpression of *hey1* mRNA at 96 hpf by WISH. **g** Expression of HSPC marker *runx1* in control embryos, *nlrc3* mutants, *nlrc3* mutants with overexpression of *nlrc3* mRNA, treatment group with JSH23, treatment group with JSH23 with overexpression of *nlrc3* mRNA at 36 hpf by WISH. **h** Expression of HSPC genes, Notch genes and genes in the downstream of *hey1* in control embryos, *nlrc3* mutants and *nlrc3* mutants with overexpression of *hey1* mRNA at 48 hpf by qPCR. ns = 0.2636, 0.2743, **P = 0.0074, 0.0014, 0.0037, ***P = 0.0007, 0.0002, 0.0004, ****P < 0.0001. **i** Enrichment plot of the NF-kB pathway relative to differentially regulated genes in the *nlrc3* morphant group compared to the control group by GSEA (Gene Set Enrichment Analysis). Normalized enrichment score = NES. P = 0.99. The statistical tests were one-sided and adjustments were made for multiple comparisons. **j** Model figure of *nlrc3* and its upstream and downstream pathways regulating zebrafish EHT. Error bars, mean ± s.d., by using two-way ANOVA with Tukey's post hoc test, *n* = 3 biological replicates in **a**, **b**, **c**. **h**. Scale bars, 100 μm in **d**, **e**, **f**, **g**. Illustrations created with BioRender.com. Source data are provided as a Source Data file.

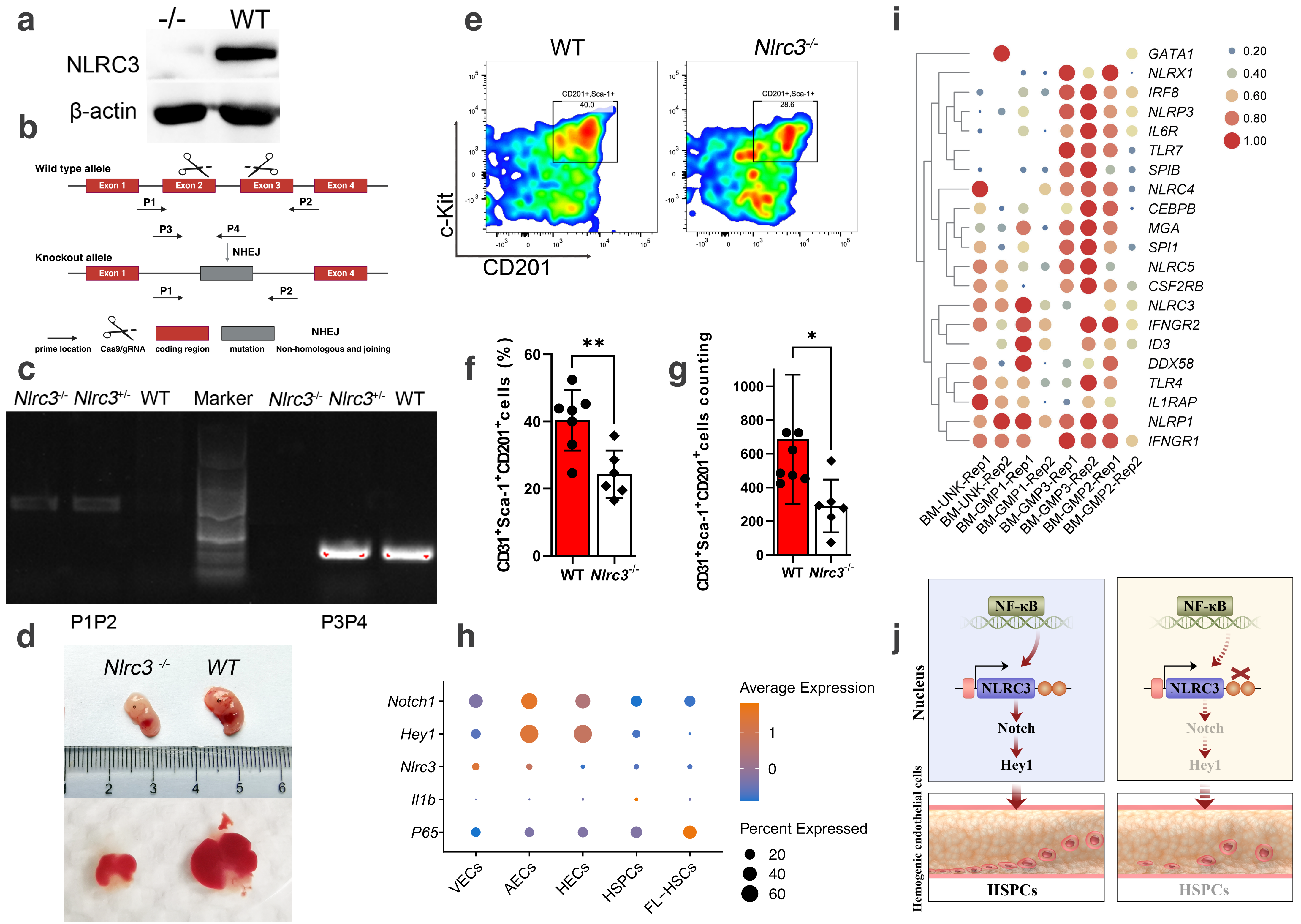

a
-/-
WT
NLRC3
β-actin
b
Wild type allele
Exon 1
Exon 2
Exon 3
Exon 4
P1
P2
P3
P4
NHEJ
Knockout allele
prime location
Cas9/gRNA
coding region
mutation
Non-homologous and joining
c
Nlrc3-/- Nlrc3+/- WT Marker Nlrc3-/- Nlrc3+/- WT
P1P2
P3P4
d
Nlrc3 -/-
WT
e
WT
Nlrc3-/-
CD201+,Sca-1+
40.0
28.6
c-Kit
CD201
f
CD31+Sca-1+CD201+cells (%)
**
WT
Nlrc3-/-
g
CD31+Sca-1+CD201+cells counting
*
h
Notch1
Hey1
Nlrc3
Il1b
P65
VECs
AECs
HECs
HSPCs
FL-HSCs
Average Expression
Percent Expressed
i
GATA1
NLRX1
IRF8
NLRP3
IL6R
TLR7
SPIB
NLRC4
CEBPB
MGA
SPI1
NLRC5
CSF2RB
NLRC3
IFNGR2
ID3
DDX58
TLR4
IL1RAP
NLRP1
IFNGR1
BM-UNK-Rep1
BM-UNK-Rep2
BM-GMP1-Rep1
BM-GMP1-Rep2
BM-GMP3-Rep1
BM-GMP3-Rep2
BM-GMP2-Rep1
BM-GMP2-Rep2
j
Nucleus
NF-κB
NLRC3
Notch
Hey1
Hemogenic endothelial cells
HSPCs

Supplement Figure 10. *Nlrc3* signaling was conserved in the regulation of vertebrate hematopoiesis.

a Protein level of *Nlrc3* in mouse embryonic mutants at E10.5. **b** Schematic illustration of *Nlrc3* knockout mice construction and verifying the mutant type. **c** DNA fragments of mutant types are separated and visualized by gel electrophoresis. **d** The photo of E14.5 fetal mice and fetal liver of *Nlrc3*$^{-/-}$ and wild-type. **e** Flow cytometry results showing the number of $CD31^{+}Sca\text{-}1^{+}CD201^{+}$ cells in the AGM of *Nlrc3* embryos decreased. **f** Percentage of $CD31^{+}Sca\text{-}1^{+}CD201^{+}$ cells. **P = 0.0026. **g** Quantification of $CD31^{+}Sca\text{-}1^{+}CD201^{+}$ cells. *P = 0.0370. **h** Bubble plot of published RNA-Seq data demonstrating the expression of *Nlrc3*, Notch and NF-kB genes in the clusters of venous endothelial cells (VECs), arterial endothelial cells (AECs), pre-hemogenic endothelial cells (pre-HECs), hemogenic endothelial cells (HECs), HSPCs, fetal liver (FL)-derived HSPCs in the mouse embryos. **i** Bubble plot of published scRNA-Seq data demonstrating the expression of *Nlrc3*, inflammatory factors, myeloid-promoting cytokines, and myeloid genes in three distinct types of granulocyte-macrophage progenitors (GMPs) derived from human bone marrow-derived $CD34^{+}$cells. **j** Schematic illustration of the role of the NLCR3 signal during EHT in vertebrates. Error bars, mean ± s.d., by using two-tailed, unpaired student's *t*-test. *n* = 8, 6 in **f**, **g.** Illustrations created with Photoshop. Source data are provided as a Source Data file.

Table S1. The Morholinos used in this work		
MO	sequence（5'-3'）	Reference
nlrc3-spMO	TTATCCTGGGTACTGAATATACCTT	This work
Control-MO	GeneTools	*Espin-Palazon R, Stachura DL, Campbell CA, et al. Cell. 2014;159(5):1070-1085.

Table S2. The primers in qPCR assay		
species	Primer name	qPCR primer sequence (5’→3’)
zebrafish	*nlrc3*-F	ACTGCCACGCCTTGTTATCT
	nlrc3-R	AAGTGTGGTGAGGGTTCGGT
	runx1-F	GCCTCTCTGCAGAACTTTCC
	runx1-R	GACGGCAGAGTAGGGAACTG
	cmyb-F	TGATGCTTCCCAACACAGAG
	cmyb-R	TTCAGAGGGAATCGTCTGCT
	Ikaros-F	AGAAGGGTAACCTGCTCCGACAC
	Ikaros-R	GGGCTTTCCAACCGAATGAGT
	il7r-F	TACACCAAACATCCCACA
	il7r-R	TCACTCACTGACGCACTT
	rag1-F	AGAGGACAGTGGGTAAAGA
	rag1-R	ATGGGTTCAGGTGTTGGTT
	lck-F	AGATGAATGGTGTGACCAGTGTA
	lck-R	GATCCTGTAGTGCTTGATGATGT
	gata1a-F	TCTGAGCCTTCTCGTTGGG
	gata1a-R	CTCTGGACGCTGGTGGAATA
	l-plastin-F	TGTCTGTGCCCGACACCAT
	l-plastin-R	GGCGGAGGCAGAGTTCAG
	pu.1-F	AGGAGTGTATGAGAGACCACATCAG
	pu.1-R	ATTTCGCAGAAGGTCAAGCA
	csf1r-F	TCCGGGACTCTCGAGTTCTCT
	csf1r-R	CTTCACCTTCTTTCCGCACATA
	lyz-F	GTGAAAATGGACGGGCTGAA
	lyz-R	CTTTGTTTGCGCTGCTCACA
	dll4-F	ACTCTTCTGCTACGGTATGTTT
	dll4-R	CAATGCTGGTTGAAGGTTTT
	ephrinB2a-F	CAAGGACAGCAAATCGAATG
	ephrinB2a-R	TGAGCCAATGACTGATGAGG
	deltaC-F	ATTATTGCACCGAACCCA
	deltaC-R	AAAATAGACCGCCCCAAC
	kdrl-F	ACTTGCCCAGATTATGGTGATG
	kdrl-R	TCAGCCTATCGTAGAGGTATCTTGTG

	il1b -F	AGAATGAAGCACATCAAACC
	il1b -R	AATCCACCACGTTCACTTC
	p65-F	CATTCCCTACGGCTAAACGA
	p65-R	AGAAAAAGGAGGTGGGTGGA
	nfkbiaa-F	AGTCATGCCAGAGAGCGAAT
	nfkbiaa-R	CAGAGCCGGATGTCATCATA
	nfkbiab-F	CAGCTCGGCGCAGATATAA
	nfkbiab-R	TCGATGAGAAGTTTGACCATGT
	nfkb1-F	CGCAAGTCCTACCCACAAGT
	nfkb1-R	ACCAGACTGTGAGCGTGAAG
	nfkb2-F	CATATGTCCCACACAATCAAGAC
	nfkb2-F	AGCCACCATAATGATCTGGAA
	notch1a-F	CGGGCCTGACGGATTCAC
	notch1a-R	GGACTCCAGCAGACGTTTAGC
	dla-F	GCAACTGAGGTGTAATGGGC
	dla-R	CACCTAAGGTTTCGAGCAGC
	dlb-R	GATGTTGCAGTCTCGCGTCC
	dlb-F	TGTTGCAGTTGGTGGCTTCG
	dlc-F	ACGAGCAGTGTGTGTAA
	dlc-R	TGTTATTCTCTGTTGACTGG
	dld-F	AGTACTGCACAGAACC
	dld-R	TCTTGGTTACAGAAGAG
	dll4-F	GCATTTTTCACGACCGTTTT
	dll4-R	CGGAAGAAAGTCCTGCAGTC
	jag1a-F	TAAATGTCCAGAAGATTACG
	jag1a-R	ACACACTGGAAGAGATTAGC
	jag1b-F	CTAGTTGCTTGTTGGTAAAC
	jag1b-R	AAGGCAGTCTACAGTGTTTC
	jag2-F	GCTCTGGATCTACCGACGTT
	jag2-R	GTCCCAAGCTTCAAGGATGA
	hey1-F	TTTGATGCTCACGCTCTGGC
	hey1-R	ACCTGCTGAGATGGGACAAG
	hey2-F	ATTGATGTGGGCAGCGAGAA
	hey2-R	TGGGATGTGGTGGATGTGGA
	her15.1-F	CACGTCACTTATTCTCATCG
	her15.1-R	TCCACAGGAGTTCAACATTG
	edn1-F	TGCGTCTACTTTTGCCACCT
	edn1-R	CCCTGTCTGACGCTGCTTTA
	hand2-F	GACGCCAAAGAAGAAAGGCG
	hand2-R	TCAGCTCCAATGCCCAAACA
	hsp70l-F	CCGCTGACTAGGGCACAT
	hsp70l-R	GACGGCATCTTTATTCACATTA
	β actin-F	GCTGTTTTCCCCTCCATTGTT

	β actin-R	TCCCATGCCAACCATCACT
	ef1a-F	GAGAAGTTCGAGAAGGAAGC
	ef1a-R	CGTAGTATTTGCTGGTCTCG

species	Primer name	qPCR primer sequence (5’→3’)
Human	*TLR4a-F*	AGACCTGTCCCTGAACCCTAT
	TLR4a-R	CGATGGACTTCTAAACCAGCCA
	NLRC3-F	GTGCCGACCGACTCATCTG
	NLRC3 -R	GTCCTGCACTCATCCAAGC
	NLRP3-F	GATCTTCGCTGCGATCAACAG
	NLRP3-R	CGTGCATTATCTGAACCCCAC
	GAPDH-F	TCCCACTCTTCCACCTTCGATGC
	GAPDH-R	GGGTCTGGGATGGAAATTGTGAGG
species	Primer name	qPCR primer sequence (5’→3’)
MOUSE	*Notch1-F*	GATGGCCTCAATGGGTACAAG
	Notch1-R	TCGTTGTTGTTGATGTCACAGT
	Notch2-F	GAGAAAAACCGCTGTCAGAATGG
	notch2 -R	GGTGGAGTATTGGCAGTCCTC
	Hey1-F	GCGCGGACGAGAATGGAAA
	Hey1-R	TCAGGTGATCCACAGTCATCTG
	GAPDH-F	AAGCGCCCTTGTGAGGAAAC
	GAPDH-R	GGTAGTTGTCGGTGAATTGGAC
	Hey2-F	GATGGCCTCAATGGGTACAAG
	Hey2-R	TCGTTGTTGTTGATGTCACAGT
	Hes5-F	AGTCCCAAGGAGAAAAACCGA
	Hes5 -R	GCTGTGTTTCAGGTAGCTGAC
	Runx1-F	ATGCCTGATCGTACCGAGAAG
	Ruxn1-R	GTCGTTGGCGTAAATGAGCTG
	GAPDH-F	TCCCACTCTTCCACCTTCGATGC
	GAPDH-R	GGGTCTGGGATGGAAATTGTGAGG

**Table S3. The zebrafish transgenic strain used in this work**		
Tg(*runx1*:EGFP;*kdrl*:mCherry)	Double-positive cells are marked the emerging HSPCs from haemogenic endothelium.	PMID: 28869969
Tg(*cmyb*:EGFP;*kdrl*:mCherry)	Item	Item
Tg(*CD41*:GFP)	Positive cells mark the HSPCs and platelets in the CHT.	PMID: 25416946
Tg(*tp1*:EGFP)	EGFP cells reflect Notch activity.	PMID: 19595765
Tg(*fli1a*:EGFP)	Positive cells mark zebrafish blood vessels.	PMID: 12167406

Tg(*kdrl*:mCherry)	Positive cells mark zebrafish blood vessels.	PMID: 28869969
Tg(*lck*:EGFP)	EGFP cells mark T-cells.	PMID: 30855581
Tg(*mpeg*:GFP)	EGFP cells mark macrophages.	PMID: 31909502
Tg(*lyz*:DsRed)	DsRED expression under the neutrophil specific promoter lysozyme.	PMID: 33162327
Tg(*mpx*:EGFP)	EGFP expression under the neutrophil specific promoter myeloperoxidase.	Item
Tg(*gata1a*:DsRed)	dsRED expression under the specific promoter erythroid transcription factor Gata1.	Item
Tg(*lcr*:EGFP)	EGFP expression under the erythroid specific globin regulatory sequence locus control region.	Item

**Table S4. Deposited Data**		
Nlrc3 knockdown RNA-seq	This work	PRJCA009166
scRNA-seq in zebrafish	Xia J, et al. Proc Natl Acad Sci USA. 2021 Apr 6; 118(14): e2015748118.	GSE146404
scRNA-seq in mouse	Zhu Q, et al. *Blood* 2020 Aug 13;136(7):845-856.	GSE137117
scRNA-seq in human	Calvanese V, et al. Nature 2022 Apr;604(7906):534-540.	GSE162950
bulk RNA-Seq in human CD34+ bone marrow $CD34^+$	Buenrostro JD, et al. *Cell* 2018 May 31;173(6):1535-1548.e16.	GSE96811

**Table S5. The antibodies used in this work**	
anti-mouse TER-119 (clone: TER-119)	BD Biosciences #560509
anti-mouse CD31-APC (clone: mmab)	BD Biosciences #551262
anti-mouse CD45-FITC (clone: OX-1)	BD Biosciences #554877
anti-mouse CD41-PE/cy7 (clone: HIP8)	BD Biosciences #561424
anti-mouse c-Kit-PE/cy5 (clone: 2B8)	Biolegend #105810
anti-mouse Sca-1-APC (clone: D7)	Biolegend #108112
anti-mouse CD45.1-AF-700 (clone: A20)	BD Biosciences #110724,
anti-mouse CD150-FITC (clone: TC15-12F12.2)	Biolegend #115902
anti-mouse CD48-PE (clone: HM48-1)	Biolegend #103406
anti-mouse CD201-APC (clone: RCR-16)	Biolegend #141506
anti-mouse β-ACTIN	Sangon Biotech #D191047
anti-mouse Anti-Nlrc3	Abcam #ab77817
